# Shaking Alone Induces De Novo Conversion of Recombinant Prion Proteins to β-Sheet Rich Oligomers and Fibrils

**DOI:** 10.1371/journal.pone.0098753

**Published:** 2014-06-03

**Authors:** Carol L. Ladner-Keay, Bethany J. Griffith, David S. Wishart

**Affiliations:** 1 Department of Computing Science, University of Alberta, Edmonton, Alberta, Canada; 2 Department of Biological Sciences, University of Alberta, Edmonton, Alberta, Canada; 3 National Institute for Nanotechnology, Edmonton, Alberta, Canada; INRA, France

## Abstract

The formation of β-sheet rich prion oligomers and fibrils from native prion protein (PrP) is thought to be a key step in the development of prion diseases. Many methods are available to convert recombinant prion protein into β-sheet rich fibrils using various chemical denaturants (urea, SDS, GdnHCl), high temperature, phospholipids, or mildly acidic conditions (pH 4). Many of these methods also require shaking or another form of agitation to complete the conversion process. We have identified that shaking alone causes the conversion of recombinant PrP to β-sheet rich oligomers and fibrils at near physiological pH (pH 5.5 to pH 6.2) and temperature. This conversion does not require any denaturant, detergent, or any other chemical cofactor. Interestingly, this conversion does not occur when the water-air interface is eliminated in the shaken sample. We have analyzed shaking-induced conversion using circular dichroism, resolution enhanced native acidic gel electrophoresis (RENAGE), electron microscopy, Fourier transform infrared spectroscopy, thioflavin T fluorescence and proteinase K resistance. Our results show that shaking causes the formation of β-sheet rich oligomers with a population distribution ranging from octamers to dodecamers and that further shaking causes a transition to β-sheet fibrils. In addition, we show that shaking-induced conversion occurs for a wide range of full-length and truncated constructs of mouse, hamster and cervid prion proteins. We propose that this method of conversion provides a robust, reproducible and easily accessible model for scrapie-like amyloid formation, allowing the generation of milligram quantities of physiologically stable β-sheet rich oligomers and fibrils. These results may also have interesting implications regarding our understanding of prion conversion and propagation both within the brain and via techniques such as protein misfolding cyclic amplification (PMCA) and quaking induced conversion (QuIC).

## Introduction

Prion protein (PrP) is a highly conserved membrane-bound protein that is particularly abundant in the neuronal cells of vertebrates. While the physiological function of properly folded and processed PrP is not yet clear, it is now clear that misfolded PrP can cause a variety of fatal neurodegenerative diseases in both animals and humans. These include scrapie in sheep, bovine spongiform encephalopathy (BSE) in cattle, chronic wasting disease (CWD) in cervids, as well as Kuru, Creutzfeld Jacob Disease (CJD) and Fatal Familial Insomnia (FFI) in humans [1]. Prions cause disease by converting from a native, helix-rich cellular form (PrP^c^) to an infectious β-sheet rich form (PrP^sc^) that is insoluble, protease resistant and highly pathogenic [2]. An abundance of misfolded PrP^sc^ proteins on the neuronal cell surface or within endosomes leads to the accumulation of extracellular amyloid protein deposits that eventually lead to cell death and the manifestation of neuronal disease. To better understand this physiological process, a number of cell-free, *de novo* methods have been developed that allow recombinant (rec) PrP^c^ to be converted to a β-sheet rich isoform. In these methods the conversion of recPrP^c^ to a β-sheet isoform is performed through the addition of denaturants and cofactors such as urea, copper ions, acid, nucleic acids, lipids and lipopolysaccharides [3], [4], [5], [6], [7], [8], [9], [10], [11]. Of course, conversion of PrP alone is not sufficient to cause prion diseases or to create infectious prion particles. Another critical component of prion disease is the occurrence of template directed replication of the infectious PrP^sc^ isoform [2], [12]. Several cell-free, *in vitro* systems have been developed that not only convert but also propagate or amplify infectious PrP^sc^ molecules. These include protein misfolding cyclic amplification (PMCA) [13], [14], [15] and quaking-induced conversion (QuIC) [16]. In these prion amplification methods, small amounts of prions (PrP^sc^) are added to large amounts of native PrP^c^ (recombinant or brain-derived) and the mixed samples are shaken or sonicated for days. Over time this mixing and high energy input leads to template-directed conversion of the native PrP^c^ to infectious prions (PrP^sc^). This conversion can be serially propagated (repeatedly adding small amounts of seed PrP^sc^ to large amounts of native PrP^c^) to generate detectable amounts of infectious prions over days or weeks [14], [16]. The shaking/sonication is thought to be necessary to provide sufficient energy to allow native prion proteins to overcome the large energy barrier associated with misfolding in a reasonable amount of time (days vs. decades). The shaking/sonication is also thought to disintegrate large fibrils, thereby generating small, oligomeric “seeds” needed to act as templates for PrP^sc^ propagation [2], [17]. In both PMCA and QuIC a number of cofactors or co-solvents must be added for the conversion and propagation of infectious prions to occur. For amplification of brain-derived prions, these include the addition of detergents such as Triton X-100 and SDS [15], [16], whereas *de novo* generation of misfolded prions, also requires additional cofactors including 1-palmitoyl-2-oleoyl-sn-glycero-3-phosphoglycerol (POPG) and RNA [18], or urea and guanidine HCl [19]. Furthermore, propagation of *de novo* generated prions requires cofactors such as 1,2-dioleoyl-sn-glycero-3-phosphoethanolamine (DOPE), in addition to Triton X-100 [20], [21].

Ideally it would be useful to develop *de novo* prion conversion and propagation methods that could be performed using only naturally occurring chemicals or naturally accessible conditions. After all, prion diseases develop in animals at physiological pH and physiological temperatures without SDS and Triton X-100. Given that the common feature of both PMCA and QuIC involves aerated shaking or agitation, and given that agitation is something that occurs naturally in all living systems, we decided to systematically explore the effects of shaking or agitation on prion conversion and propagation *in vitro*.

Here we demonstrate that shaking alone induces the conversion of recombinant PrP (recPrP) to β-sheet rich oligomers and fibrils using both full length (recPrP^c 23–231^) and truncated PrP (recPrP^c^ ^90–231^) constructs. These PrP isoforms share aspects of both the proteinase K (PK) resistant PrP^sc^ isoform and the spontaneously generated amyloid isoform. We have found that shaking of recPrP at near physiological pH (5.5 to 6.2), without any denaturants or cofactors, causes conversion to β-sheet rich oligomers and fibrils. We have characterized these oligomers and fibrils using resolution enhanced native acidic gel electrophoresis (RENAGE), circular dichroism (CD), electron microscopy (EM), Fourier transform infrared spectroscopy (FTIR), PK resistance and thioflavin T (ThT) fluorescence. During shaking-induced conversion the monomers disappear as oligomers appear and then disappear, with fibrils forming in their place. The formation of fibrils shows a sigmoidal growth as shown by the fluorescence enhancement of ThT and RENAGE. We also show that the propagation of fibrils occurs by seeding with shaking-induced fibrils. In addition, we show that shaking-induced conversion occurs for a wide range of full-length and truncated constructs of mouse, hamster and cervid prions. We propose that this method of conversion can generate milligram quantities of physiologically stable β-sheet rich oligomers and fibrils. The cofactor free nature of this conversion method makes it applicable to the screening of potential small molecule prion inhibitors. Furthermore our results have interesting implications in protein misfolding cyclic amplification (PMCA) and quaking induced conversion (QuIC), where agitation-induced conversion may occur incidentally.

## Materials and Methods

### RecPrP^c^ purification

Truncated recombinant prion proteins from Syrian hamster, mouse and white-tailed deer (cervid) constructs (recShPrP ^90–232^, recMoPrP ^90–231^, recMoPrP ^120–231^ and recCePrP ^94–233^) with His6x-tags were expressed in *E. coli* and purified as previously described [9], [22]. In addition, a full-length recMoPrP ^23–231^ construct was generated similarly by inserting MoPrP ^23–231^ into a pET15b expression plasmid containing an N-terminal fusion tag attached to a His6x tag, a thrombin cleavage site and an enterokinase cleavage site (MGSSHHHHHHSSGLVPRGSHMDDDD). All constructs were purified on Ni-NTA (Qiagen Canada, Toronto, Canada) as previously described [22] and a protease inhibitor cocktail (Roche Diagnostics, Indianapolis, USA) added to eluted PrP fractions at a dilution of between 100X and 25X, from a tablet dissolved in 1 mL of water. In addition, for the full-length MoPrP ^23–231^, 1 mM EDTA was added to the eluted PrP fractions. Protein samples were then dialyzed first into 50 mM sodium acetate pH 5.5, followed by 18Ω water and then lyophilized. The purity of all constructs was confirmed to be greater than 98% by SDS-PAGE. The protein concentration was determined using absorption extinction coefficients determined by Protparam (ExPASy) and confirmed by a Bradford protein assay (Biorad Laboratories Canada, Mississauga, Canada).

Removal of the His6x purification tag was performed with bovine thrombin (Sigma-Aldrich Canada, Oakville, Canada) at a ratio of 1∶2000 in 10 mM Tris pH 7.1 for 6 hrs. The reaction was then stopped by removing thrombin with Pierce SBTI-agarose (Thermo Scientific, Rockford, USA) or p-aminobenzamidine-agarose (Sigma-Aldrich Canada) and then applying the sample to a PD10 desalting column equilibrated in 50 mM sodium acetate pH 5.5. Additionally, 0.1 mM PMSF was added to the desalted sample which was then dialyzed into water, snap frozen and lyophilized.

### Shaking-induced conversion of recPrP to oligomers and fibrils

Selected samples of recPrP constructs from each species were shaken under various conditions to convert them to a β-sheet rich isoform. Standard conditions for conversion of all PrP constructs involved using 0.4 mL of a 0.5 mg/mL recPrP^c^ solution in 20 mM sodium acetate, pH 5.5 with 0.02% sodium azide, being shaken at either 350 or 250 rpm at 37°C in a 1.5 mL polypropylene MaxyClear microcentrifuge tube (Corning Life Sciences –Axygen, Union City, USA). Different shaking speeds had to be used because the orbital incubator used at 350 rpm broke and was replaced with a shaker with a different orbital diameter. Samples shaken at 350 rpm, were placed on a Lab-line 3527 orbital shaker with a 0.75′′ orbit diameter; whereas, samples shaken at 250 rpm were placed on a Lab-line Orbit 3590 shaker with 2′′ orbit diameter. We found that shaking recMoPrP ^90–231^ and recShPrP ^90–232^ at 350 rpm with a 0.75′′ orbit was equivalent to shaking at 250 rpm with a 2′′ orbit. This was determined after achieving a similar distribution of oligomers (8–12 mers), high molecular weight oligomers (16–20 mers) and fibrils by RENAGE. Conversion was also performed by shaking samples at room temperature with recMoPrP ^90–231^ at 350 rpm on a Lab-line 4626 orbital shaker (0.75′′ orbit). The tube was taped horizontally onto an orbital platform for shaking, because more extensive conversion was found in this case than for a vertically fixed tube. For conversion at pH 6.2 the buffer was 10 mM MES and 5 mM sodium acetate with shaking being performed at 350 rpm at 37°C. For conversion at pH 7.4 the buffer was 20 mM sodium phosphate with shaking being performed at 350 rpm at 37°C.

The effects of the shaking speed on conversion were tested on the orbital shakers with different orbit diameters. Shaking recShPrP ^90–232^ at 200 rpm with a 0.75′′ orbit diameter for 3 days resulted in no conversion as seen by RENAGE. It is not known if longer periods of shaking would have resulted in the formation of oligomers. However, we also tested the shaking threshold for conversion on the 2′′ orbit shaker. In this case, no conversion is found when shaking recMoPrP ^90–231^ at 75 rpm for up to 4 days. When shaking recMoPrP ^90–231^ at 150 rpm we found essentially no conversion after 3 days, then <1% oligomerization/aggregation after 4 days and <2% oligomerization after 7 days of shaking (result not shown). However shaking recMoPrP ^90–231^ at 200 rpm on the 2′′ orbit shaker does generate oligomers and fibrils of a similar distribution to that seen at 250 rpm. We also tested shaking induced conversion on an incubator with an orbit diameter of 1′′ (Lab-line 3520). For this incubator we found that shaking in the range from 250 to 350 rpm was necessary to generate oligomers and fibrils (results not shown).

For comparison we generated oligomers from recMoPrP ^90–231^ and recShPrP ^90–232^ using more conventional prion conversion conditions: 3 M urea, 20 mM sodium acetate, pH 4 and 200 mM NaCl [4]. We also generated prion fibrils using standard conditions [23] by shaking recMoPrP ^23–231^ with a protein concentration of 1 mg/mL in 1 M guanidine HCl, 3 M urea, 50 mM HEPES and 150 mM NaCl, pH 7 at 350 rpm and room temperature for 3 days. Fibrils were then dialyzed into 20 mM sodium acetate pH 5.2 for further analysis.

Sonication of recMoPrP ^90–231^ and recMoPrP ^23–231^ was conducted on an Ultrasonic 3000 Homogenizer (BioLogics Inc., Manassas, VA, USA) with a 3.8 mm micro tip or a 12.7 mm tapped tip, as indicated. Buffer conditions and prion concentrations were the same as used for shaking-induced conversion. For sonication using a micro tip, the end of the tip was placed directly in the prion protein solution. For sonication with the regular, tapped tip, the end of the probe was placed in the water bath, immediately at the side of a 0.2 mL PCR tube containing the prion sample. To mimic PMCA-like sonication, prion samples were sonicated for 1.2 minutes (2 minutes, pulsed at 60%) every 30 minutes for 24 hrs, in a 0.2 mL thin-walled PCR tube.

### Resolution Enhanced Acidic Gel Eletrophoresis (RENAGE)

The size of the prion monomers, oligomers and fibrils were analyzed using a specially developed technique called RENAGE and sized via a PrP PICUP ladder (a ladder consisting of different covalently linked PrP oligomers). Gels were prepared using a 8% acrylamide pH 4.3 running gel and a 3% acrylamide pH 5.2 stacking gel as previously described [22]. The running buffer consisted of 0.35 M β-alanine and 0.14 M acetate at pH 4.3. Gels were pre-run at 30 mAmp per gel for 20 minutes and then 5 µg of the prion sample was loaded in dissolving buffer. The dissolving buffer contains 37% glycerol, 128 mM acetate-KOH, pH 5.2 and 0.01% crystal violet (Sigma-Aldrich Canada). The gels were run at 30 mAmp for 75 to 85 minutes with reverse polarity. Gels were stained with colloidal coomassie blue for approximately four hours and destained in water [24]. The PrP PICUP ladder was generated by cross-linking recMoPrP^c^ ^90–231^ at 1 mg/mL by photo-induced cross-linking of unmodified protein PICUP [25] as previously described [22].

The time course of shaking-induced oligomer and fibril formation process was determined by removing 20 µL of sample (MoPrP ^90–231^ or MoPrP ^23–231^) from each 0.4 mL sample of shaken recPrP at different time points. Samples were then stored in a refrigerator until the completion of time course study and then the RENAGE analysis was performed. Quantitation of monomers, oligomers and fibrils was performed by first scanning the gel image and then converting the gel lanes to a band-intensity chromatogram using ImageJ (http://rsbweb.nih.gov/ij/index.html). Chromatograms were then plotted using the Origin software package (OriginLab Corp., version 9) and the peaks manually marked and integrated, using Origin's “peak analyzer” module. Percentages of each oligomer class were then calculated from the area of each peak compared to the total integrated area. The RENAGE fibril peak areas were plotted versus time and fitted to a sigmoidal function (y = a/(1+exp(-k*(x-xc))) or exponential function (y = A1*exp(x/t1)+y0) using the Origin software package.

### Circular Dichroism

The secondary structure of each PrP construct and PrP oligomers of recShPrP ^90–232^ and recMoPrP ^90–231^ was determined using CD. Spectra were acquired on a Jasco J-810 circular dichroism spectropolarimeter in a 0.1 mm quartz cell with samples dissolved in 20 mM sodium acetate, pH 5.5 or water at pH 5.5. Spectra were recorded as the average of three scans from 190 to 260 nm, acquired with a scan rate of 20 nm/min and smoothed with a Savitzky–Golay window of 9 or 11 points. The secondary structure was determined using CDPro [26] with the CONTINLL program [27] using the SP22X reference protein set.

### Fourier Transform Infrared Spectroscopy (FTIR)

FTIR spectra were acquired on a Varian FTS-7000 infrared spectrometer (Varian) equipped with a DTGS (deuterated triglycine sulfate) detector. MoPrP^c 23–231^, MoPrP^ 23–231^ oligomers and MoPrP ^23–231^ fibril samples (50 µL of 2–3 mg/mL) were dried onto a CaF_2_ plate under nitrogen. Spectra were acquired from 96 scans at a sensitivity of 2 and a resolution of 2 cm^−1^. First a background scan was run with a CaF_2_ plate in place and then a blank spectrum was acquired with buffer dried onto the CaF_2_ plate. Spectra were smoothed with a Savitsky-Golay window of 9 points using the Varian Resolution Pro software. FTIR spectra were further processed using Origin (Version 9) to assist with spectral deconvolution and secondary structure quantification. The second derivatives of the smoothed spectra were calculated using Origin (Version 9) with 2^nd^ order Savitsky-Golay smoothing and a 9-point window. Before deconvolution, the baseline was subtracted from each absorbance spectrum. Absorbance spectra were then deconvoluted using Origin's multiple peak fit, employing non-linear Lorentz curve fitting by fixing the wavenumber of the peak maxima from the second derivative spectra and using a peak width of 20 cm^−1^. The deconvoluted spectrum was then fit to multiple Gaussian curves by fixing the peak wavenumbers using the second derivative of the deconvoluted spectra and fixing the peak width to 13 cm^−1^.

### Negative stain electron microscopy

PrP converted isoforms were applied to UV irradiated 300 mesh copper grids with a support film of Formvar with carbon (Ted Pella, Inc., Cat# 01753-F, Redding, CA, USA). The samples were diluted to 2 µM PrP in water and 10 µL of the prion solution was spotted onto Parafilm. The grid was placed on top of the droplet for 1 minute. Grids were washed once with 10 µL water and stained two times with 10 µL of 4% uranyl acetate, whereby each time the residual solution was wicked away using filter paper. Micrographs were acquired on a Philips/FEI (Morgagni) transmission election microscope.

### Thioflavin T fluorescence time course study

The progression of PrP^c^ conversion to fibrils during shaking was monitored by Thioflavin T (ThT) fluorescence enhancement. MoPrP ^90–231^ and MoPrP ^23–231^ were converted by shaking at 250 rpm and 37°C as described above. PrP samples were then removed at different time points and mixed with 20 mM sodium acetate pH 5.5 and 100 µM ThT (Sigma) to give 10 µM PrP (monomeric concentration). Emission spectra were acquired on a QuantaMaster 400 spectrofluorimeter (Photon Technology International Inc., London, ON, Canada) with an excitation wavelength of 440 nm. The fluorescence intensity at the emission maximum of 480 nm was then plotted over the entire sampling period. The ThT fluorescence time course was fit to a sigmoidal function as described above for the RENAGE fibril peak area.

### Proteinase K digestion

The susceptibility/resistance to PK digestion was tested for recMoPrP^c^, shaking-induced recMoPrP ^23–231^ fibrils and sonicated recMoPrP ^23–231^ samples using a method adapted from Atarashi *et al.* [15]. For digestion, samples at 0.5 mg/mL PrP in 20 mM sodium acetate pH 5.5 were diluted 2 fold into 50 mM Tris pH 8. Then proteinase K (Promega, Madison, WI, USA) was added at ratios of 1∶400, 1∶200 and 1∶50, PK to PrP (g:g). The sample was digested for 1 hr at 37°C. After digest, ∼4 M urea was added to individual samples along with Laemmli sample buffer (2% SDS, 5% glycerol, 2 mM DTT, 50 mM Tris, pH 6.8, 0.01% bromophenol blue) and boiled at 95°C for 5 min. Samples were ran on a 12% SDS-PAGE gel with a Tris-Glycine buffer, pH 8.8 system and visualized with colloidal coomassie blue.

## Results

### Shaking alone induces recPrP^c^ to form β-sheet rich oligomers

**Figure 1:**
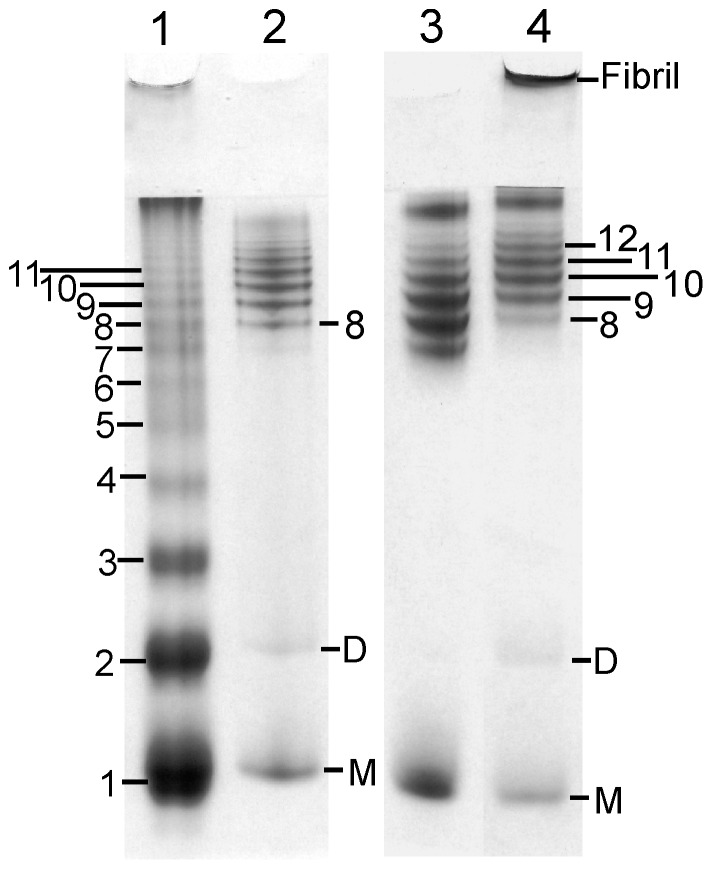
RENAGE indicates that shaking recPrP^c^ generates oligomers. A PrP PICUP ladder (lane 1) is used to size the oligomers formed by shaking recMoPrP ^90–231^ in pH 5.5 buffer at 350 rpm and 37°C for 1 day (lane 2). Shaking-induced oligomers are predominantly a distribution of 8-mers to 13-mers. In comparison oligomers formed in urea and salt exhibit a bimodal size distribution of 7-mers to 12-mers (lane 3). Longer periods of shaking recMoPrP ^90–231^ (shaking at 350 rpm, 37°C for 2 days) will also generate a fibril band and bimodal distribution of 8-mers to 12-mers and larger oligomers (>16-mers) (lane 4).

RENAGE [22] was initially used to discover the formation of oligomers induced by shaking recShPrP ^90–232^ and recMoPrP ^90–231^ at 37°C and 350 rpm, in buffer at pH 5.5. The size of these recMoPrP ^90–231^ oligomers follows a Gaussian or near-Gaussian distribution ranging from octamers to dodecamers as determined using a specially prepared PrP PICUP ladder (Fig. 1). This PrP PICUP ladder is generated by non-specifically cross-linking MoPrP ^90–231^ using the PICUP reagent [22]. The same size and distribution of shaking-induced oligomers was produced from shaking recShPrP ^90–232^ (results not shown). Interestingly, this size distribution is distinct from what is seen when oligomers of heptamers and larger are formed using more conventional techniques: 3 M urea and 200 mM NaCl at pH 4 [22] (Fig. 1). Shaking the PrP oligomer samples for longer times caused the formation of larger oligomers (>16 mers) and a protein band that barely enters the stacking gel as seen for recMoPrP ^90–231^ (marked as the fibril band, Fig. 1, lane 4). This result is also seen for all recPrP constructs that we studied (results not shown). Formation of these shaking-induced oligomers occurs with or without the addition of 150 mM NaCl (results not shown). This is in contrast to urea induced conversion, where conversion to oligomers is dependent on the presence of NaCl [28].

**Figure 2:**
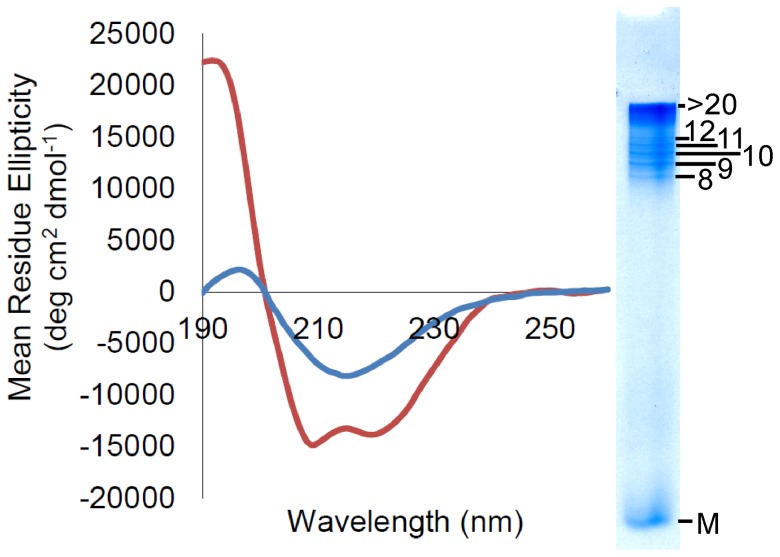
Circular dichroism (CD) indicates that shaking-induced oligomers contain significant quantities of β-sheet. Shaking recShPrP ^90–232^ at 350 rpm (in pH 5.5 water and 150 mM NaCl) induces conversion from an α-helical protein (red line) to a β-sheet rich structure (blue line). The inset, on the right, shows the corresponding RENAGE gel of the same sample, indicating a preponderance of oligomers. CDPro analysis for native PrP^c^ gives 43% α-helix and 10% β-sheet, and for oligomers it yields 16% α-helix and 24% β-sheet.

CD was used to analyze oligomers that were formed by shaking recShPrP ^90–232^ and recMoPrP ^90–231^ and confirmed by RENAGE. This showed that ShPrP ^90–232^ oligomers have a β-sheet structure (Fig. 2). An aliquot of the same sample used for CD was analyzed by RENAGE and exhibited a bimodal size distribution with smaller oligomers (octamers to dodecamers) and larger oligomers (>16-mers), but no or little fibril band. Secondary structure content of these shaking-induced oligomers was found to be 25% β-sheet and 12% α-helix for recShPrP ^90–231^ as calculated by CDPro. A similar CD spectrum was acquired for a shaken recMoPrP ^90-231^ sample with approximately equal abundance of oligomers and fibrils (by RENAGE). These MoPrP^90–231^ oligomers were also found to be β-sheet rich (24% β-sheet from CDPro). In contrast, monomeric recShPrP^c^ ^90–232^ and recMoPrP^c^ ^90–231^ contained 42% α-helix and 10% β-sheet. This result is consistent with previous findings that the appearance of discreet oligomer bands seen by RENAGE match with having a β-sheet structure [22].

**Figure 3:**
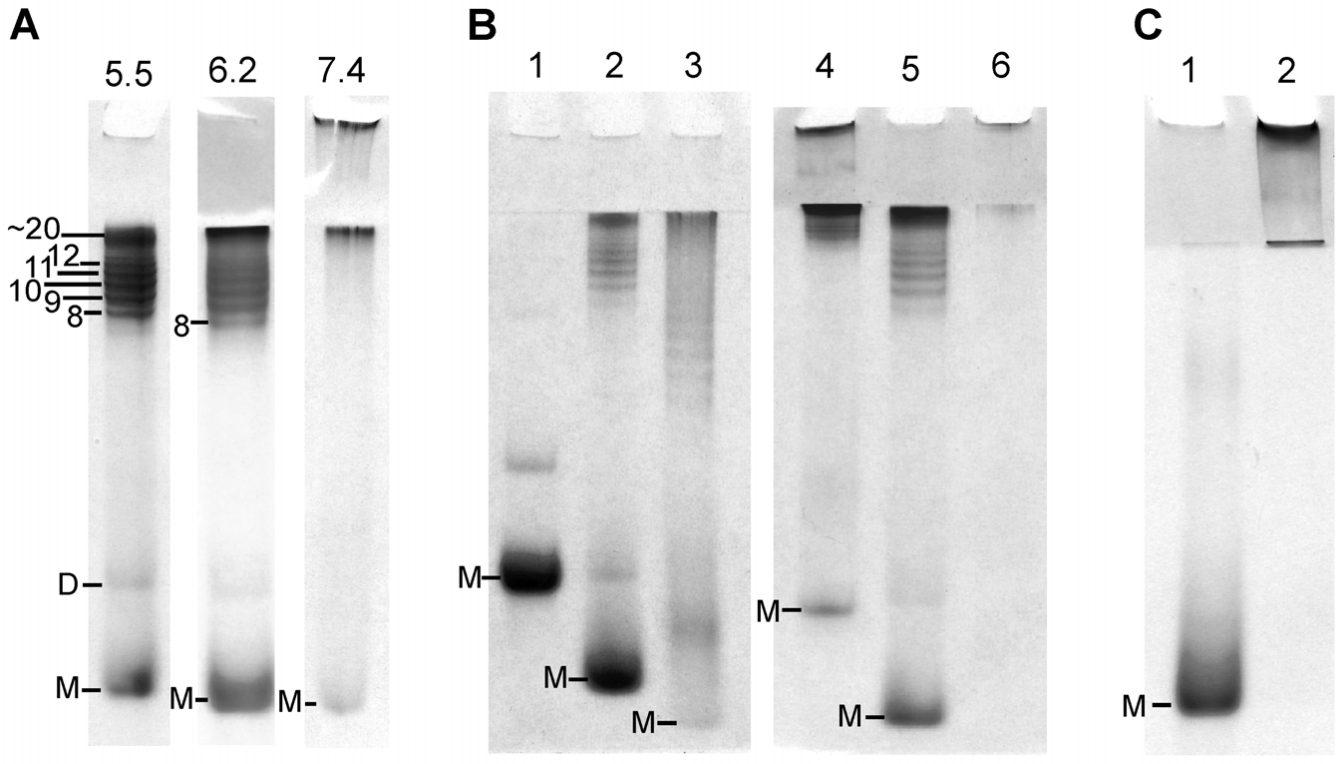
RENAGE of shaking-converted prions under various conditions. A) ShPrP ^90–232^ oligomers are formed by shaking in pH 5.5, 6.2 and 7.4 buffers, at 350 rpm and 37°C. B) RENAGE after shaking at 350 rpm for 1 day with full length recMoPrP ^23–231^ (lane 1), truncated recMoPrP ^90–231^ (lane 2) and C-terminal domain recMoPrP ^120–231^ (lane 3) and after 2 days with recMoPrP ^23–231^ (lane 4), truncated recMoPrP ^90–231^ (lane 5) and C-terminal domain recMoPrP ^120–231^ (lane 6). Samples in panel B were shaken at 350 rpm and 37°C in pH 6.2 buffer. C) Shaking a 0.6 mL solution of recShPrP^90–232^ in a 0.6 mL centrifuge tube without any air or bubbles for two weeks (lane 1) as compared to the same sample of 0.4 mL in a 1.5 mL centrifuge tube (i.e. with air), shaken for one week (lane 2).

Shaking-induced oligomers were also found at pH 6.2 using recShPrP ^90–232^ and recMoPrP ^90–231^ (result shown for recShPrP ^90–232^ in Fig. 3A). The distribution of oligomers formed at pH 6.2 is similar to that formed at pH 5.5. However at pH 6.2 there is an increase in high molecular oligomers (16 to 20-mers) relative to oligomers at 8 to 12-mers (Fig. 3A). Furthermore shaking-induced conversion occurred when recShPrP ^90–232^ and recMoPrP ^90–231^ were shaken at room temperature. In contrast when shaking-induced conversion was tested at pH 7.4 the oligomers formed are predominantly large oligomers (>16-mers) and included a large oligomer band referred to as a fibril band on the RENAGE gels. Prion protein constructs of different lengths for MoPrP were assessed for their ability to convert to oligomers after 24 and 48 hrs shaking at pH 6.2, using RENAGE. Shaking-induced conversion occurs for full length recMoPrP ^23–231^ in a manner similar to truncated recMoPrP ^90–231^ although apparently with different kinetics (Fig. 3B). In contrast, shaking the C-terminal domain (recMoPrP ^120–231^) causes faster conversion as seen at 24 hrs and then after 48 hrs only large oligomers are visible by RENAGE (Fig. 3B). In this recMoPrP ^120–321^ sample which was shaken for 48 hours, there is a loss in the total amount of protein deposited on the gel in addition to the formation of a visible precipitate in the sample tube. This could indicate that after (C-terminal domain) oligomers form they preferentially progress to aggregates rather than fibrils. Conversion of these three different lengths of MoPrP occurred similarly at pH 5.5, except the C-terminal (recMoPrP ^120–231^) low molecular weight oligomers (ie. 8-mers) were not as distinct. We proceeded to characterize shaking-induced conversion at pH 5.5, because of the efficiency of forming oligomers for recMoPrP ^90–231^ and recMoPrP ^23–231^ at this pH and because the sodium acetate buffer was more amenable to CD analysis than the buffer containing MES. It is also notable that shaking-induced conversion occurs irrespective of the presence of the His6x purification tag (Fig. S1), with oligomers of the same size formed with or without the His6x tag. We also converted cervid PrP ^94–233^ to oligomers and fibrils, as seen by RENAGE (results not shown).

The formation of these shaking-induced oligomers requires an air-water interface. This was shown by the lack of oligomerization when a 0.6 mL sample of 0.5 mg/mL recShPrP ^90–232^ was placed in a 0.6 mL centrifuge tube, and shaken at 250 rpm and 37°C, for two weeks (Fig. 3C). It is important that the air-water interface was eliminated by filling the tube, such that no air bubbles were present. Shaking recShPrP ^90–232^ also in a completely filled tube (hence no air bubbles) at 350 rpm and 37°C, also remained monomeric as seen by RENAGE. Furthermore CD analysis of the same sample, shaken with no air-water interface, showed that there was no conversion to a β-sheet structure. All of the results presented in this paper were from shaking-induced conversion performed with a 1.5 mL centrifuge tube place on its side (unless otherwise stated). Experiments were conducted in this manner because it was found that conversion occurred faster when the tube was on its side, rather than when it was placed upright on a shaking platform (result not shown). This increase in conversion speed could be due to an increase in the water-air surface area.

**Figure 4:**
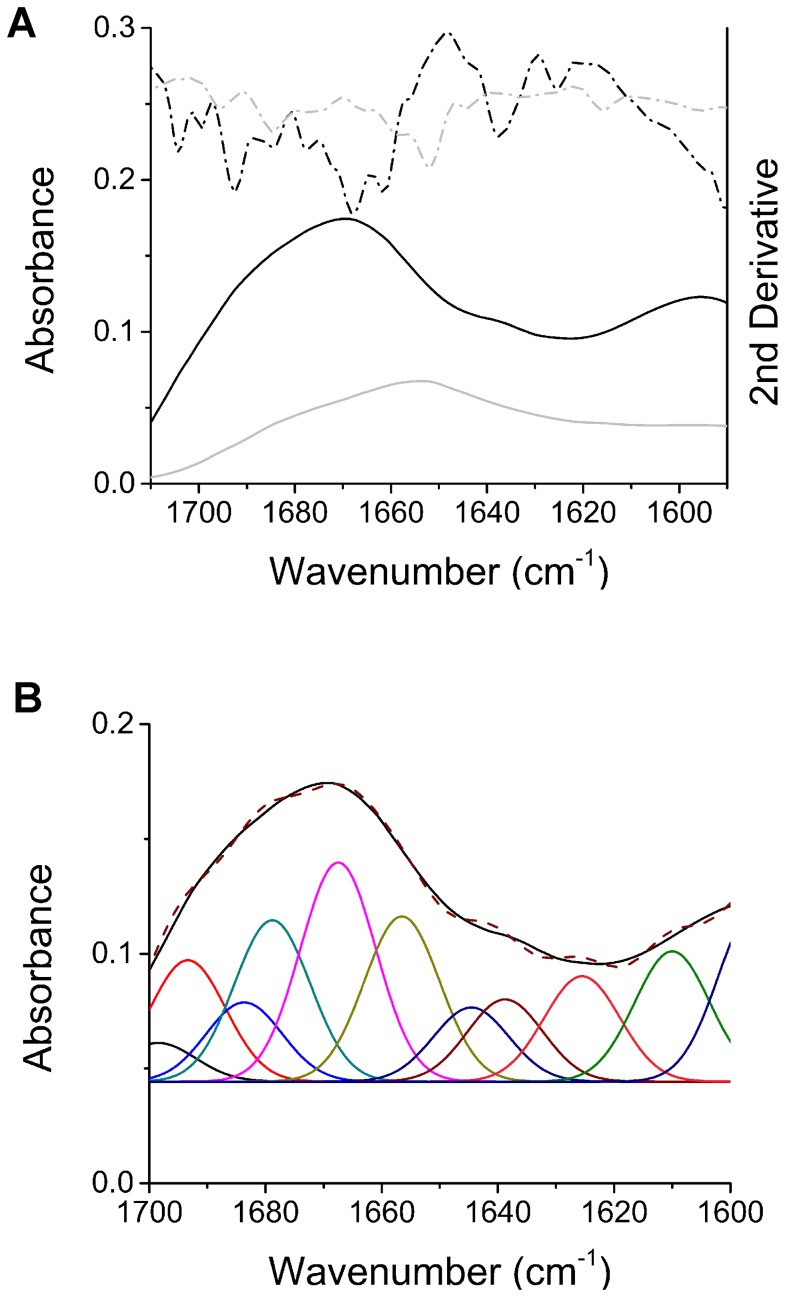
Fourier transform infrared spectroscopy shows that shaking-induces conversion to oligomers with increased β-sheet structure, dominated by turns and loops. A) FTIR of oligomers formed by shaking-induced conversion (at 250 rpm and 37°C) of recMoPrP ^23–231^ (black line) is drastically different from monomeric recMoPrP^c^ ^23–231^ (grey line). The absorbance spectra are shown in solid lines and the corresponding 2^nd^ derivative spectra are shown in dashed lines. B) Spectral deconvolution and component analysis of the fibril FTIR spectrum (solid line) is fit with Gaussian peaks to a deconvoluted spectrum (dashed line).

In addition to CD analysis of ShPrP ^90–232^ and MoPrP ^90–231^ oligomers, the FTIR of the amide I band was used to characterize MoPrP ^23–231^ oligomers. The full-length construct was used so that we could focus on the characterization of the more physiologically relevant full-length recMoPrP ^23–231^ construct. The FTIR spectrum is shown for an oligomer sample from 0.4 mg/mL recMoPrP ^23–231^ shaken at 250 rpm and 37°C for 3 days (Fig. 4). The FTIR spectrum along with its second derivative of the oligomer sample shows the presence of different peaks compared to those found for the predominantly α-helical recMoPrP^c^ ^23–231^. Spectral deconvolution was performed on the FTIR absorbance spectra to determine the secondary structure composition of these oligomers (Fig. 4B). The areas of the resulting Gaussian peaks seen in Fig. 4B were used to determine the structural content (Table 1). The FTIR data shows that the recMoPrP ^23–231^ sample transitioned from 33% α-helix in the recPrP^c^ sample to only 16% α-helix in the oligomeric sample. In its place the structure of the oligomers has transitioned to 18% β-sheet with significant turn and loop peaks (∼1662 and 1679 cm^−1^).

**Table 1:** Secondary structure composition of shaking-induced oligomers as determined from deconvolution and curve fitting of the FTIR amide I band.

Assignment	Wavenumber (cm^−1^)	Secondary structure (%)
Intermolecular β-sheet	1626	10
β-pleated sheets	1639	8
Random coil	1645	7
α-helix	1657	16
Turns	1662 to 1668	21
Turns/loops	1679	16
Anti-parallel β-sheet/turn	1684	7.8
Anti-parallel β-sheet	1693	12

In this study shaking-induced conversion was tested on three different shaking incubators. Initially a shaker with a 0.75′′ orbit diameter was used at 350 rpm. In experiments, where indicated, a shaker with a 2′′ orbit diameter was used at 250 rpm. These two shaking speeds used with their respective orbit diameters (350 rpm; 0.75′′ orbit and 250 rpm; 2′′ orbit) generated oligomers of a similar distribution between oligomers and fibrils. To assess the reproducibility of shaking conversion with other orbit radii we also shook recMoPrP ^90–231^ with a 1′′ orbit diameter. When comparing shaking speeds of 200 rpm, 300 rpm and 350 rpm we found that shaking at all three speeds generated the typical bimodal oligomer pattern, with more low molecular weight oligomers (8 to 12-mers) than high molecular weight oligomers (16 to 20-mers). Conversion to fibrils proceeded the fastest at 300 rpm with complete conversion after just 4 days, based on RENAGE (results not shown).

### Characterization of shaking-induced β-sheet fibrils

**Figure 5:**
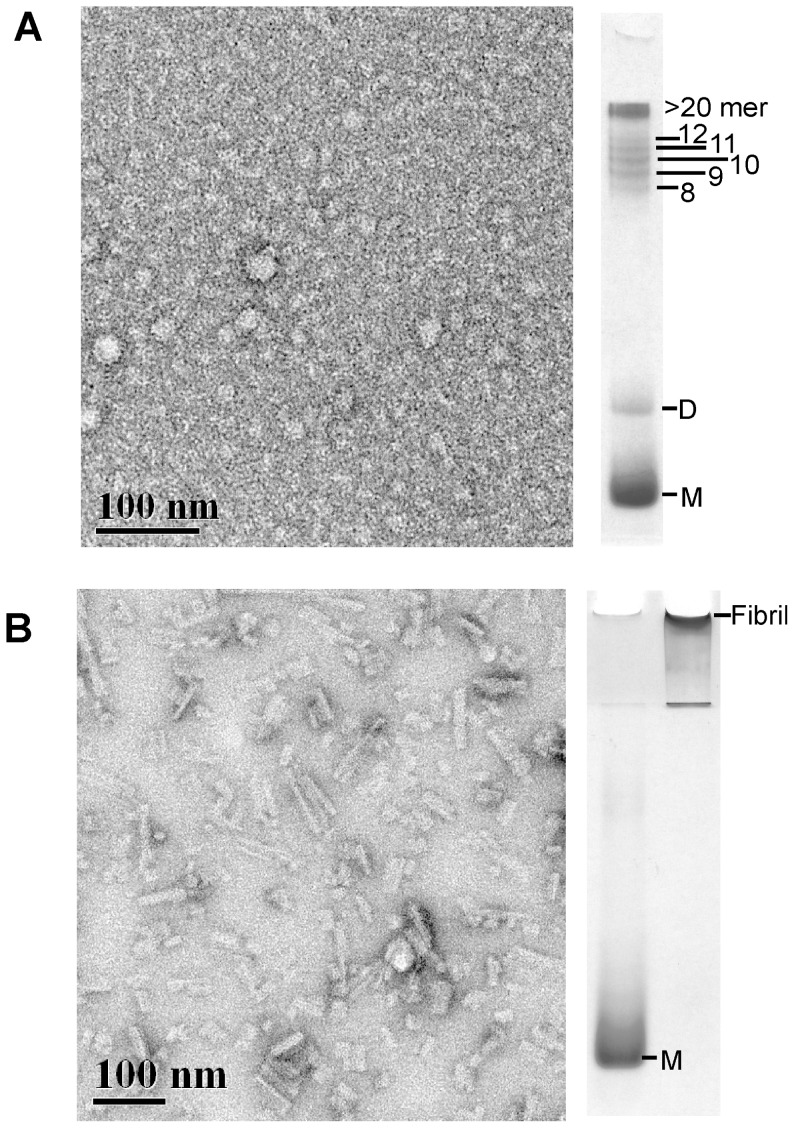
Electron microscopy confirms the formation of oligomers and fibrils seen in RENAGE. Negative stain EM of shaking-induced prion oligomers (panel A) and fibrils (panel B). The oligomers shown here were formed from shaking recMoPrP ^90–231^ at 350 rpm at room temperature for 1 day. The fibril sample was formed by shaking recShPrP ^90–232^ at 350 rpm at 37°C for 5 days. The corresponding RENAGE analysis of the same sample is shown alongside the micrograph. The indicated scale bar = 100 nm.

To characterize the progression of PrP monomers to prion fibrils throughout the shaking period, we analyzed the shaking-converted isoforms by negative stain electron microscopy (EM). These samples were generated using different shaking conditions and different time points to provide an oligomer sample free of fibrils and a fibril sample free of oligomers. A sample of prion oligomers was generated by shaking recMoPrP^c 90–231^ monomers at 350 rpm, at room temperature for 1 day. The sample was shown by RENAGE to contain only oligomer bands and no fibril band. The sample was shaken at room temperature to enrich for oligomers and avoid the formation of fibrils, which was routinely found when shaking recPrP at room temperature, rather than 37°C. EM analysis of this sample showed that the oligomers were ∼20 nm disc-like structures (Fig. 5A). It should be noted that there is an enrichment of high molecular weight oligomers (∼20-mers) in this sample that likely aided in visualizing the oligomers by EM. EM characterization also confirmed what the RENAGE analysis initially showed: that the sample contained PrP oligomers only and no detectable fibrils. In contrast, PrP^c^ that was shaken for 5 days at 350 rpm at 37°C, showed only a fibril band on RENAGE and contained abundant rod-like fibrils as seen by EM (Fig. 5B). The dominant species seen on the grid were these rod-like fibrils with no significant patches of the oligomeric structures that are seen in panel A. EM was also performed for recMoPrP ^90–231^ and recMoPrP ^23–231^ fibril samples (based on RENAGE) and showed the formation of similar rod-like fibrils (results not shown). However EM of shaking-induced conversion of MoPrP ^120–231^ did not show any rod-like fibrils, but rather only showed round clusters consistent with amorphous aggregates. However EM cannot rule out that fibrils are formed by shaking this C-terminal construct. This is because the fibrils may have been stuck to the tube and were at low abundance.

**Figure 6:**
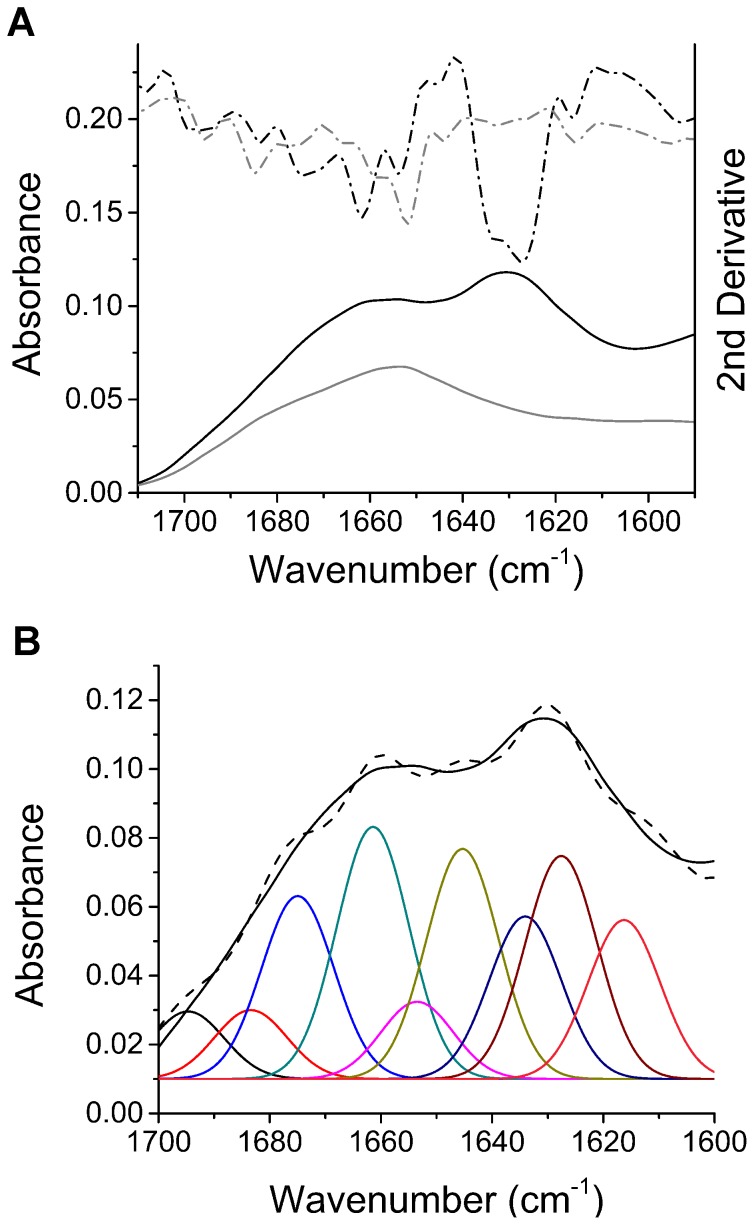
Fourier transform infrared spectroscopy shows that shaking-induced fibrils are rich in β-sheet. A) FTIR of fibrils formed by shaking-induced conversion of recMoPrP ^23–231^ (at 250 rpm and 37°C) shows they are rich in β-sheet structure (black line), as compared to monomeric recMoPrP^c^ ^23–231^(grey line). The absorbance FTIR spectra are shown in solid lines and the corresponding 2^nd^ derivative spectra are shown in dashed lines. B) Spectral deconvolution and component analysis of the fibril FTIR spectrum (black) generated by fitting Gaussian peaks to a deconvoluted spectrum (brown line).

FTIR spectroscopy was also used to characterize the fully converted, shaking-induced fibrils. The extent of their conversion and fibril content was confirmed by RENAGE. Figure 6A shows the FTIR absorbance spectra and second derivative of both the full-length, native recMoPrP^c^ ^23–231^ and the same protein fully converted to fibrils via shaking. The negative peaks seen in the second derivative spectra were used to assign the secondary structure components based on previously assigned PrP^sc^ FTIR spectra [29] and *de novo* PrP fibril FTIR spectra [30]. For the shaking-induced PrP fibrils, the prominent peaks are a 1627 cm^−1^ peak assigned to intermolecular hydrogen bonds characteristic of β-sheets and a 1634 cm^−1^ peak assigned to β-pleated sheets. In comparison, in the recMoPrP^c^ ^23–231^ spectrum the predominant peak is 1652 cm^−1^, which is the characteristic absorbance of α-helices (Fig. 6A). FTIR spectral deconvolution was used to determine the percentage of secondary structure components from the shaking-induced fibril FTIR spectrum (Fig. 6B). Table 2 shows the full peak assignment and secondary structure percentages determined from Gaussian deconvolution of the FTIR spectrum. From this spectral deconvolution analysis it was determined that shaking-induced fibrils contained 38% β-sheet and 5% α-helix (Table 2). In addition, we compared the FTIR spectrum obtained for shaking-induced fibrils with those from fibrils generated by shaking PrP^c^ in 2 M guanidine HCl (GdnHCl) and 1 M urea, pH 7 as described by Baskakov and colleagues [4], [30] (Fig. S2A). Differences between the fibrils formed by the two conversion methods are very slight, but there appears to be small differences in the amount of intermolecular hydrogen bonding (1627 cm^−1^) and intramolecular β-pleated sheet (1634 to 1637 cm^−1^). In particular the GdnHCl/urea-formed fibrils exhibit a slight increase in the amount of the intermolecular hydrogen bonding (1627 cm^−1^) compared to the shaking-induced fibrils. (Fig. S2 and Table S1).

**Table 2:** Secondary structure composition of shaking-induced fibrils as determined from deconvolution and curve fitting of the FTIR amide I band.

Assignment	Wavenumber (cm^−1^)	Secondary structure (%)
Intermolecular β-sheet	1616	11
Intermolecular β-sheet	1627	16
β-pleated sheets	1634	11
Random coil	1645	16
α-helix	1653	5.4
Turns	1662	18
Turn/loops	1675	13
Anti-parallel β-sheet/turn	1683	4.8
Anti-parallel β-sheet	1694	4.7

### Amyloid-like properties in shaking-converted prion fibrils

**Figure 7:**
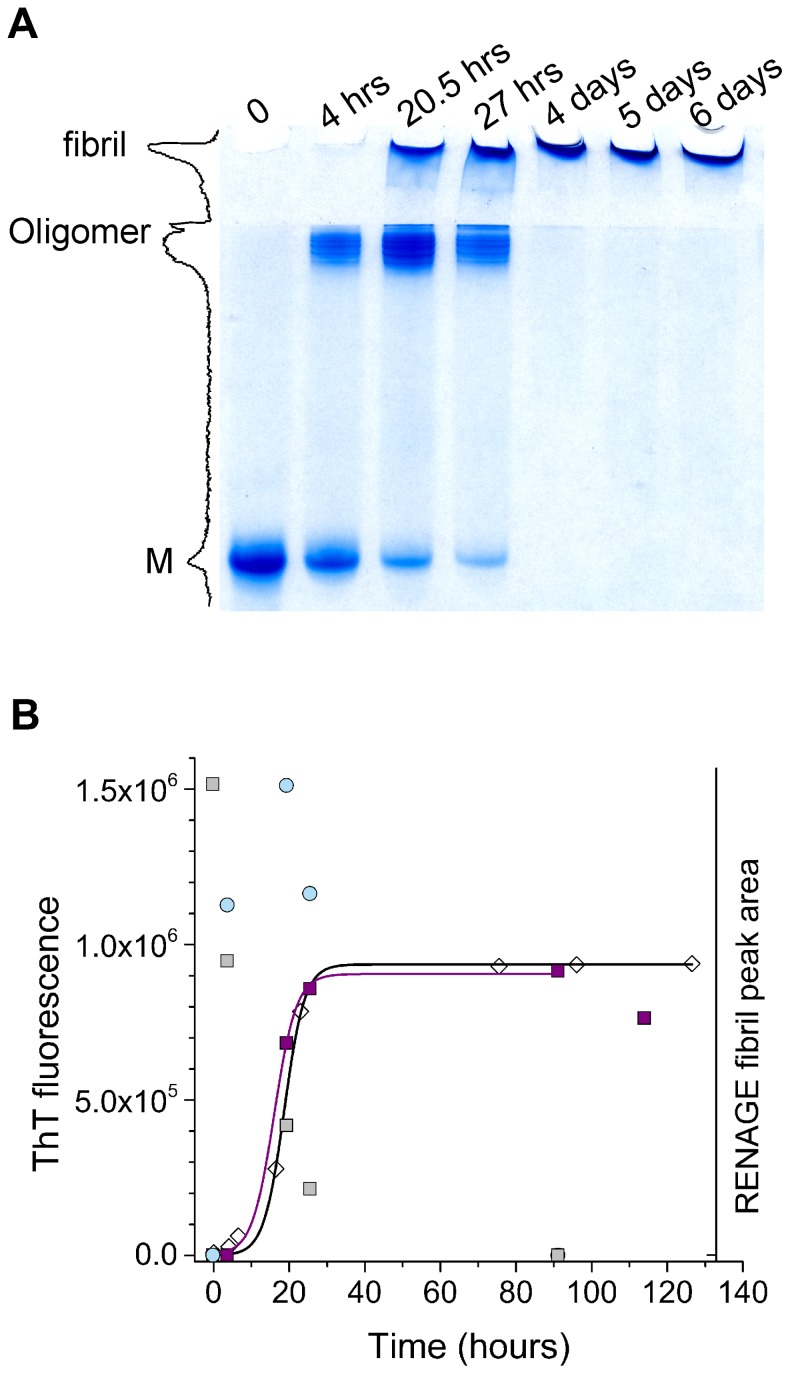
Time course of the formation of prion oligomers and fibrils. A) RENAGE gel of shaking converted recMoPrP ^23–231^ at 250 rpm and 37°C shows a time dependent loss of monomer, formation of oligomers and subsequent formation of fibrils. The chromatogram profile of each gel lane was acquired to determine the fibril content. A representative profile after 27 hours of shaking is shown. B) Plot of the time dependent thioflavin T (ThT) fluorescence (open diamonds; black line) and RENAGE fibril peak area (purple filled squares; purple line) shows a sigmoidal growth in both ThT fluorescence enhancement and fibril formation.

The time course of shaking-induced conversion of recMoPrP ^23–231^ was followed using RENAGE (Fig. 7A). This allowed us to quantify the amount of monomer, oligomer and fibril throughout the conversion process, using a single technique. As seen in Fig. 7A there is a loss of monomer that is concurrent with the formation and loss of oligomers, followed by the abrupt formation of fibrils. A time course for recMoPrP ^90–231^ also showed a loss of monomer concurrent with the formation of oligomers and a shift to fibrils (result not shown). We also used ThT to probe for the formation of the characteristic cross-β structure found in amyloids [23], [31]. Previously we determined that shaking-induced fibrils enhance ThT fluorescence (results not shown). Consequently, we monitored the time course changes in ThT fluorescence during fibril formation, by shaking alone. Plotting the time course of ThT fluorescence over time we show a sigmoidal growth in the number of fibrils (Fig. 7B). On the same plot we also show that the growth of the fibril band in RENAGE was also sigmoidal (Fig. 7B). This suggests that the RENAGE fibril band is a suitable way to follow the kinetics of PrP fibril formation. Furthermore, the ability to overlay the growth of ThT fluorescence with the RENAGE fibril band growth indicates that it is the fibrils that are responsible for the characteristic cross-β structure of PrP amyloid fibrils. The fact that the fibrils (and not oligomers) exhibit amyloid-like structure was further confirmed when we found that PrP oligomers formed by urea conversion do not enhance ThT fluorescence (result not shown).

**Figure 8:**
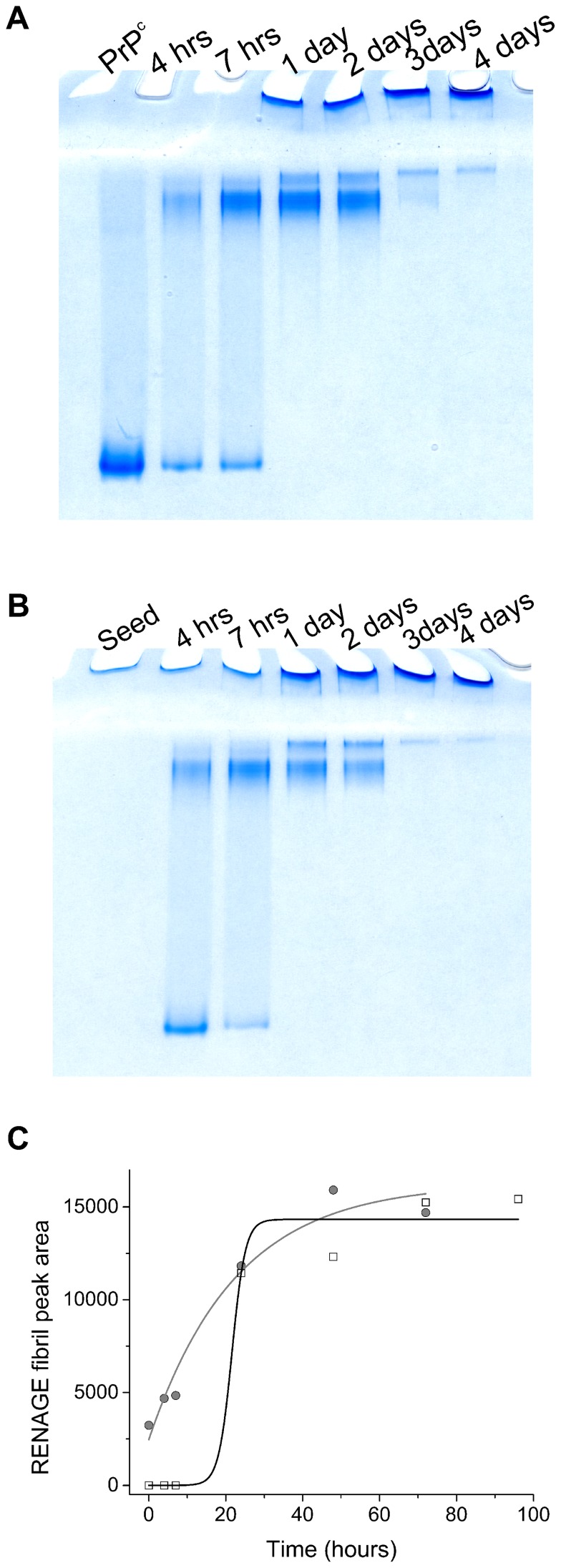
Growth of shaking-induced fibrils with seeding is exponential. A) RENAGE gel of the time course of shaking induced conversion of recMoPrP ^23–231^ at 250 rpm and 37°C. B) RENAGE gel of shaking induced conversion of the same recMoPrP (same batch) under the same conditions except with seeding using 5% MoPrP ^23–231^ fibrils into fresh recMoPrP^c^. C) The chromatogram profile of each gel lane was acquired to determine the fibril content. Time dependent fibril content growth is shown plotted against time and has a sigmoidal dependence when starting with only fresh recMoPrP^c^ ^23–231^ (open squares, black line). Upon seeding with 5% PrP fibrils the fibril content grows logarithmically (grey circles, grey line).

In addition to testing the amyloid character of shaking-induced fibrils, we also tested if shaking-induced fibrils could seed and propagate fibril growth. For this we conducted a serial dilution study where small amounts of shaking-induced fibrils were added to fresh recMoPrP^c 23–231^. These serial dilution studies showed that if the sample is not shaken, fibril formation could not be propagated upon dilution of 5% fibril into fresh recPrP^c^ (data not shown). However, if the sample was shaken, fibril formation occurred faster when fresh PrP^c^ was seeded with 5% fibrils, than if no seed was added (Fig. 8A,B). The time dependence of the fibril formation as determined from RENAGE of seeded and unseeded fibril growth was fitted to exponential and sigmoidal functions, respectively (Fig. 8C). Later time points are not shown in Fig. 8C because of a loss of fibril content after the end point of the sigmoidal growth. We attribute this to loss of sample due to either fibril-fibril aggregation or adsorption of the fibrils onto the plastic container [32]. We have repeated the propagation of fibril formation by seeding fresh PrP^c^ with the shaking-induced prion fibrils for five generations (i.e. five 1∶20 serial dilutions). During these propagation steps the kinetics seen by RENAGE did not change.

### Proteinase K resistance of shaking-induced fibrils

**Figure 9:**
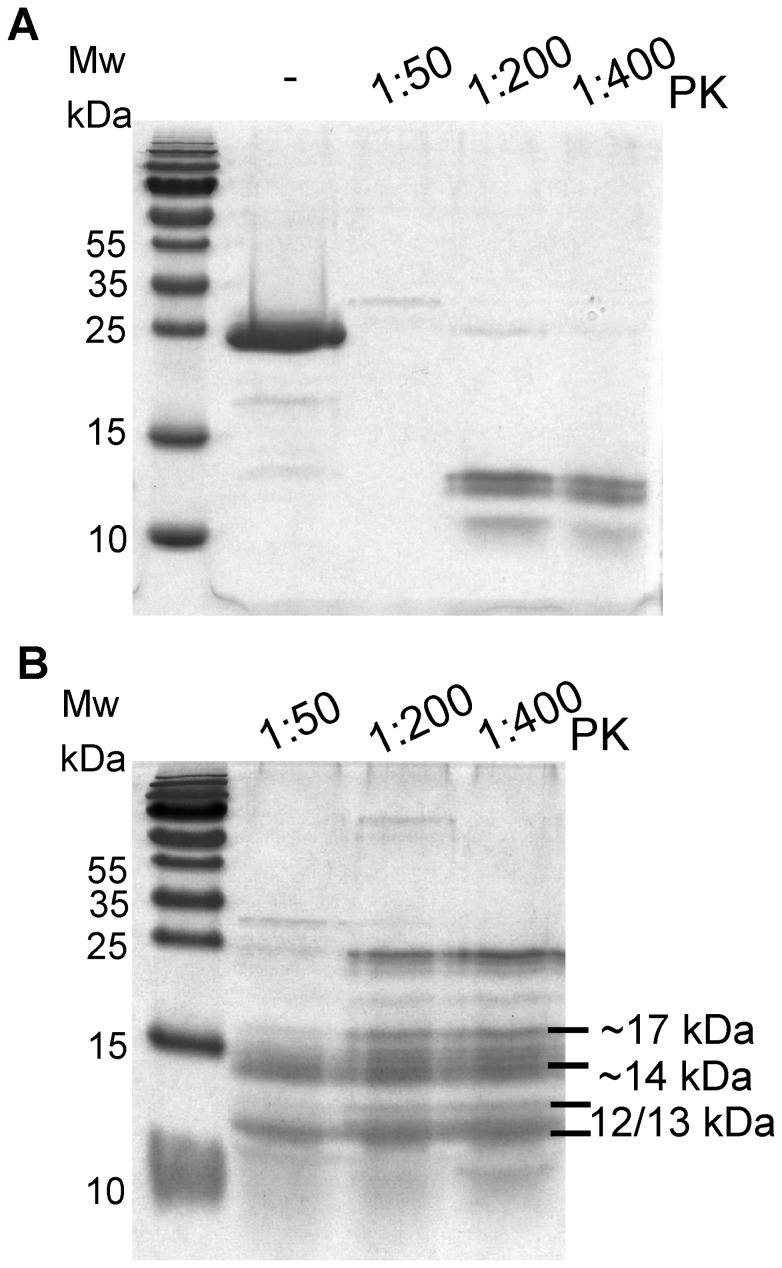
Shaking-induced fibrils have Proteinase K resistance. SDS-PAGE of recMoPrP^c^ ^23–231^ (panel A) and fibrils (panel B) without (PK-) and with PK at 1∶50, 1∶200 and 1∶400 (PK:PrP, g:g) shows that shaking-induced fibrils have 12, 13, 14 and 17 kDa resistance bands.

Naturally occurring infectious prions, as well as many *in vitro* converted fibril forms, are known to exhibit PK resistance [33], [34]. In fact, PK resistance is considered to be a hallmark for the presence of PrP^sc^. As expected, we found that shaking-induced fibrils (from recMoPrP ^23–231^) are PK resistant (Fig. 9A,B). As seen in this gel there are three bands of approximately 12, 13 and 14 kDa corresponding to PK resistant fragments. Interestingly, we also observed the presence of low levels of a ∼17 kDa PK resistance fragment. This may be the same, previously published, 17 kDa band that is found in PrP^sc^ that has been PK digested after deglycosylation [15]. In fact, a 17 kDa band is also seen in recombinant PrP^sc^ generated via PMCA and POPG/RNA that has been PK digested [18]. However in these cases, the 17 kDa fragment, from PK digested PrP^sc^, is often as abundant at the 12/13 kDa bands.

Given that the ∼17 kDa PK resistant fragment seems to be characteristic of infectious prions and given that the 12/13 kDa fragments are often found in non-infectious prions, we are now working on modifying our shaking conversion protocol to see if we can enhance the proportion of the 17 kDa fragment. This could lead to the generation of a self-propagating form similar to that described by Deleault et al., [21]. We also tested the PK resistance of fibrils generated after five serial propagations, but found that the PK resistance of the resulting fibrils did not change (results not shown).

### Sonication versus shaking

**Figure 10:**
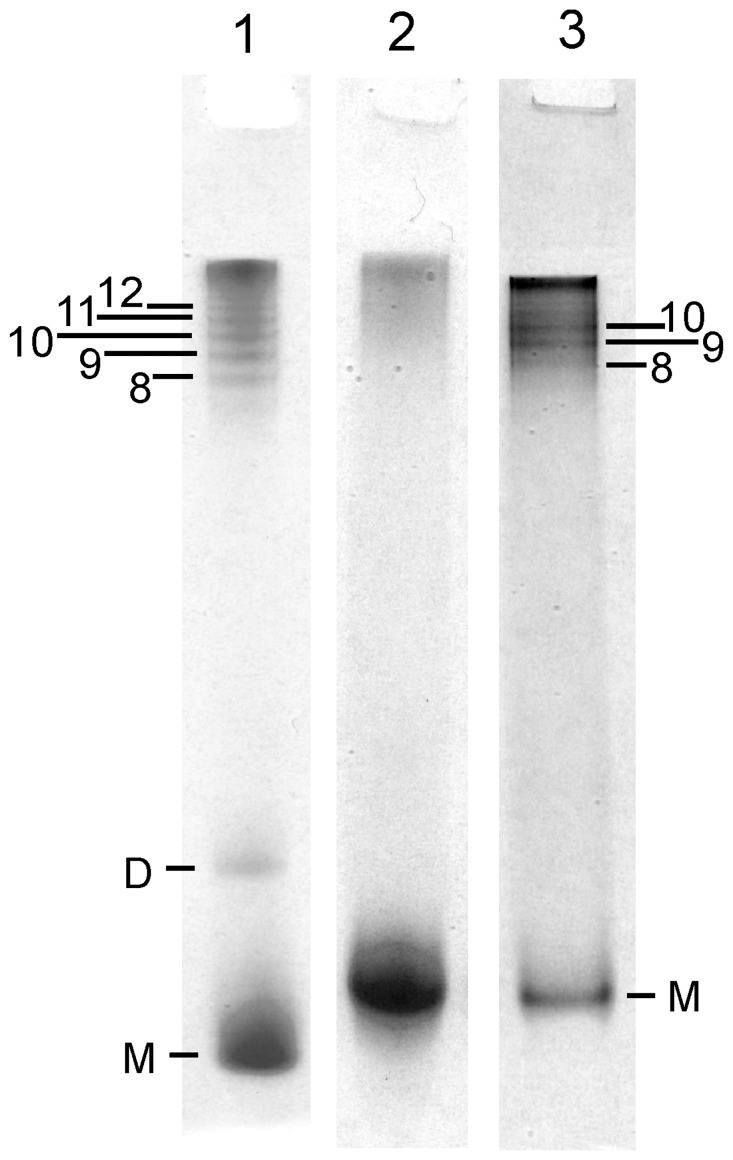
Sonication of PrP generates oligomers. RENAGE of recMoPrP ^90–231^ sonicated for 8 cycles of 10 seconds each, show that oligomers are generated (lane 1). Furthermore sonication of recMoPrP ^23–231^ in a single PMCA-like round generates oligomers (lane 2). The formation of oligomers in a PMCA-like round is enhanced by placing the probe inside the solution (lane 3).

Because sonication (as opposed to shaking) is commonly used for PMCA, we also tested the effect of sonication, alone, on oligomer formation. In our first experiment we investigated what sonication would do to a solution (0.5 mg/mL) of recPrP without the usual detergent additives of SDS or Triton X-100. Figure 10 shows that sonication (for 8 cycles of a 10 sec pulse) using a microprobe directly in the sample of recMoPrP^c^ ^90–231^ results in the formation of a mixture of large oligomers (>14-mers; 25%), 7 to 12-mers (23%) and monomers (49%). This suggests that sonication is a much more powerful and a far faster approach to prion conversion to oligomers than shaking. However, the sonication-induced conversion under these conditions does not convert all of the monomeric recPrP, even after 10 cycles of sonication (for a total sonication time of 100 sec). We also tested whether repeated sonication, using a similar scheme as in PMCA, will increase the level of prion oligomerization. We sonicated a sample of 0.5 mg/mL recMoPrP^c^ ^23–231^ at pH 5.5 in a 0.2 mL PCR tube for 2 min every 30 min over a 24-hour cycle. We found a small amount of oligomer (∼20%) formed when the sample was sonicated with the horn outside of the thin-walled PCR tube, and more oligomers (89%) were found when a micro tip was placed directly inside the tube, using a 24-hour cycle (Fig. 9). In this latter sample, sonication-induced conversion generated a sample of 51% large oligomers (>14-mers), 38% small oligomers (7 to 12-mers) and 1% fibrils, with 11% monomer remaining. We also tested for PK resistance in the sonicated recMoPrP ^23–231^ material but found that the samples were not PK resistant (data not shown). This is consistent with the very low PK resistance (in comparison to fibrils) found for β-oligomers [34]. Furthermore it indicates that the material generated from sonication, without detergents, does not generate the same prion isoform which forms spontaneously from PMCA [15].

## Discussion

Our results clearly show that shaking-alone can convert recombinant PrP^c^ to β-sheet rich oligomers and fibrils. This is the first demonstration that the conversion of native recombinant PrP to β-sheet oligomers and fibrils can occur under physiological conditions (i.e. without the addition of detergents, denaturants, low pH, or high temperatures). Previously the only other *de novo* conversion method that approached physiological conditions was a conversion protocol that used a pH 4 buffer with shaking at 8 rpm [35]. Essentially, all other *de novo* methods used to convert PrP to misfolded prions employ detergents (SDS, Triton X-100), denaturants (urea, guanidine, high temperatures, extreme pH), lipid surfactants (POPG), large cofactors (RNA), or combinations of the above [3], [4], [5], [9], [11], [15], [18], [36], [37]. Because shaking-induced conversion is free of these chemical contaminants or cofactors, we believe the characterization of misfolded prions generated by shaking-induced conversion will potentially provide a “cleaner” framework with which to understand the prion conversion and propagation process. In particular, we believe that the shaking-induced conversion protocol we have developed could be used as a new model for cell free, *de novo* PrP conversion that could be used to identify or characterize prion inhibitors. In fact, we are currently optimizing a shaking-induced conversion assay to screen known and potential prion small molecule inhibitors. All previously published cell-free assays for screening small molecule inhibitors for prion conversion used denaturants [23], [38], [39]. However, it has been noted that the presence of urea and other denaturants can significantly change the mode of action and effectiveness of small molecules in prion conversion [39]. The ability to generate *de novo* prion oligomers and fibrils under simple, physiological conditions could not only improve the accuracy of small molecule screening assays but also renew interest in finding small molecule inhibitors through cell free conversion assays [23]. Furthermore, the ability to routinely generate milligram amounts of stable oligomers and fibrils, in a simple, uncontaminated physiological buffer, will certainly enhance the opportunities for high-resolution structural and biophysical studies of prions.

It is important to distinguish our findings and our methods from some of the better-known protocols for prion conversion, propagation and detection – namely PMCA and QuIC. In PMCA, sonication is used to generate or amplify PrP^sc^ from brain-derived or recombinant PrP^c^ [13], [15]. During the initial development of PMCA, spontaneous formation of a protease resistance isoform was found to occur during the sonication of recombinant PrP^c^ in 0.1% SDS but it could be prevented by the addition of 0.1% Triton X-100 [15]. Consequently, the standard protocol for all modern PMCA methods involves the addition of Triton X-100 (a detergent). The use of detergents and extended periods of sonication in PMCA makes the technique relatively non physiological. It also prevents the technique from being used in ligand screening assays or in generating material for biophysical or high-resolution structural studies. In QuIC, a finely tuned protocol with intermittent shaking is used to amplify PrP^sc^ via the addition of external, recombinant PrP^c^ [16], [40]. With QuIC, spontaneous formation of a protease resistant PrP isoform can occur with shaking using a pH 7 buffer with 0.1% SDS and 0.1% Triton X-100 [16]. This kind of spontaneous amyloid formation can lead to false positives, which can be prevented in QuIC by using an optimized recombinant PrP^c^ concentration, an optimized sample volume and an optimized temperature [16]. Rather than preventing this spontaneous conversion to a protease resistant amyloid isoform, we have identified conditions to robustly convert recPrP^c^ to PrP^sc^ like isoforms. Overall, our demonstration that shaking, alone, can create PrP oligomers and fibrils with the characteristic biophysical features (fibril structure, β-rich, ThT binding, amyloid character, PK resistant, serially propagating) seen in prions generated by PMCA or QuIC or *in-vitro* prion detection methods has some interesting implications. In particular, our data shows that shaking PrP at higher concentrations (0.5 mg/mL) than standardly used in QuIC[16], [41], [42], can result in the spontaneous formation of β-sheet rich isoforms that exhibit PK resistance. This spontaneous/shaking-induced conversion can lead to false positives in prion detection assays.

Our observation that shaking-induced prion conversion required air or an air-water interface (Fig. 3C) suggests a possible mechanism by which the α-helical PrP^c^ is converted into a β-sheet rich isoform. In particular, the presence of an air-water interface appears to provide a denaturing (i.e. hydrophobic) environment that causes partial unfolding and clustering of the prion protein. Several reports have recently appeared describing the importance of an air-water interface in protein denaturation, in protein aggregation and in amyloid conversion for myoglobin, Aβ and insulin [32], [43], [44]. These data are consistent with our results showing that conversion does not occur when PrP is shaken without the presence of a small layer of air above the solution (Fig. 3C). While air-water interfaces are easy to generate in the laboratory, they are not particularly common physiologically. However, air-water interfaces with significant levels of turbulence and shaking are found in the stomach, the large intestine and rumen of mammals. Given that prion proteins occur throughout the body (including the gut) and that prion diseases are mostly transmitted via consumption of prion-infected material, it is not hard to imagine that the initial, infectious prion seeds could be generated in the gut prior to moving to the brain.

Another mechanism explaining prion protein conversion by shaking suggests that it is due to hydrodynamic forces caused by vortexing. Vortexing leads to protein denaturation by sheering forces as well as secondary seeding effects [45]. Previously it was found that vortexing insulin solutions causes a decrease in CD ellipticity at 210 nm, which is concurrent with the formation of an insulin amyloid [45]. A similar effect is also seen in generation of denaturant-induced (urea/guanidine HCl) prion fibrils formed with shaking [30]. These prion fibrils have a 205 nm feature that may correspond to a superhelical structure [30]. The effect of hydrodynamic forces on protein aggregation suggests that shaking-induced conversion of PrP is physiologically relevant because these forces can be found *in vivo* with biological fluids, such as blood [46] or in the rumen, stomach, or intestine of mammals.

## Supporting Information

Figure S1 **RENAGE of oligomers formed with and without a His6x tag.**
(PDF)

Figure S2 **Fourier transform infrared spectroscopy of shaking-induced fibrils compared to GdnHCl/urea formed fibrils.**
(PDF)

Table S1 **Secondary structure composition of GdnHCl/urea formed fibrils as determined from deconvolution and curve fitting of the FTIR amide I band.**
(PDF)
