# Supplementary figures and images for: Shaking Alone Induces De Novo Conversion of Recombinant Prion Proteins to β-Sheet Rich Oligomers and Fibrils
Source: PLoS One. 2014 Jun 3;9(6):e98753. doi: 10.1371/journal.pone.0098753 (PMC4043794; doi:10.1371/journal.pone.0098753)

**Figure S1. RENAGE of oligomers formed with and without a His6x tag.**

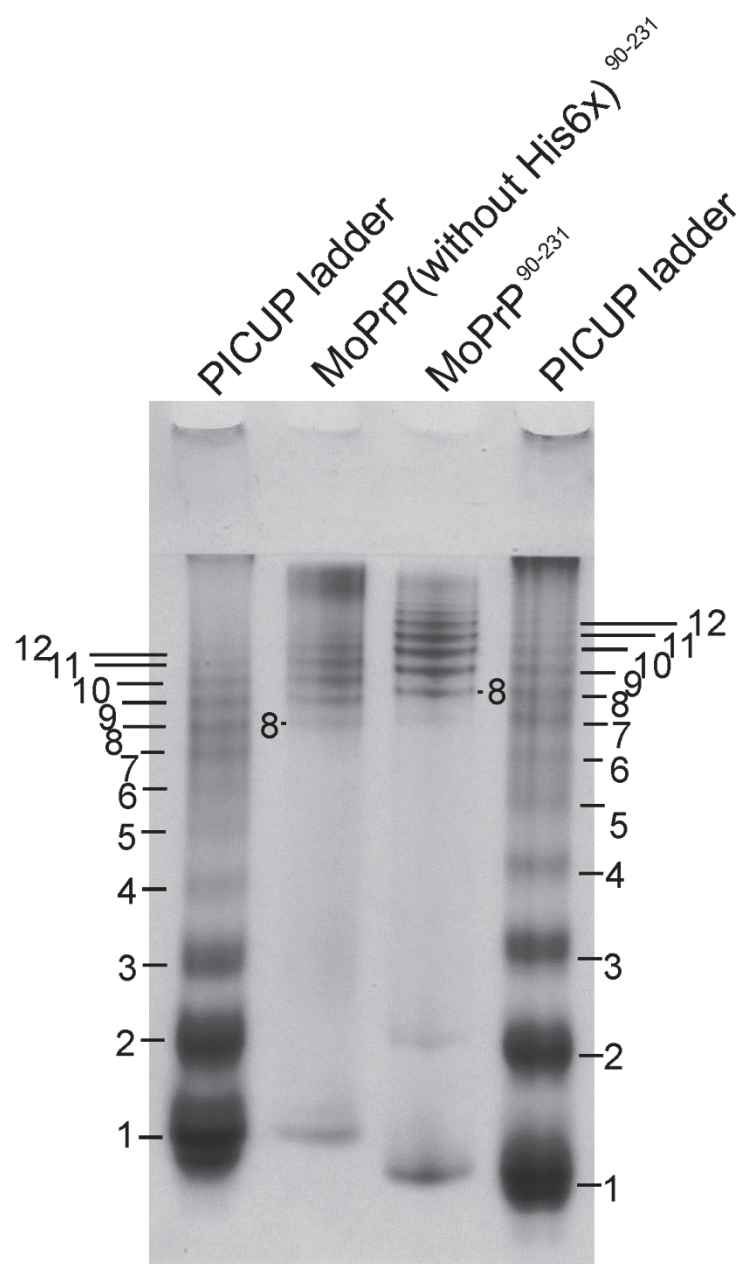

Supplement: Figure S1 — RENAGE of oligomers formed with and without a His6x tag. (PDF) [file pone.0098753.s001.pdf]
