# Supplementary material for: Shaking Alone Induces De Novo Conversion of Recombinant Prion Proteins to β-Sheet Rich Oligomers and Fibrils
Source: PLoS One. 2014 Jun 3;9(6):e98753. doi: 10.1371/journal.pone.0098753 (PMC4043794; doi:10.1371/journal.pone.0098753)

Figure S2. Fourier transform infrared spectroscopy of shaking-induced fibrils compared to GdnHCl/urea formed fibrils. A) Fourier transform infrared spectroscopy of shaking-induced prion fibrils (purple line) compared to GdnHCl/urea-formed fibrils (black line). The GdnHCl/urea-formed fibrils were generated with shaking at 350 rpm. B) Spectral deconvolution and component analysis of the fibril spectrum (black line) by fitting Guassian peaks to a deconvoluted spectrum (purple line).

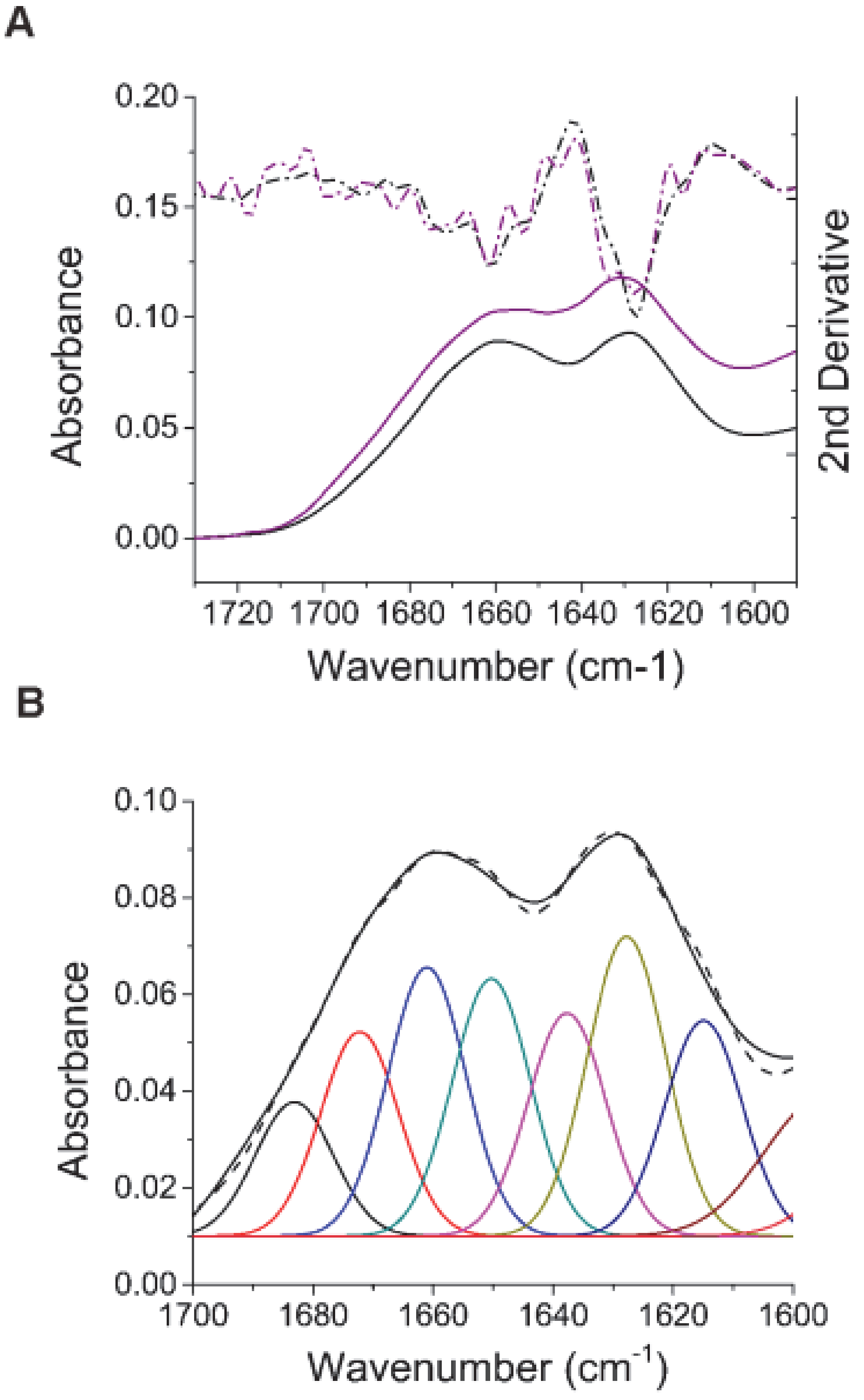

Supplement: Figure S2 — Fourier transform infrared spectroscopy of shaking-induced fibrils compared to GdnHCl/urea formed fibrils. (PDF) [file pone.0098753.s002.pdf]
