# Supplementary material for: Shaking Alone Induces De Novo Conversion of Recombinant Prion Proteins to β-Sheet Rich Oligomers and Fibrils
Source: PLoS One. 2014 Jun 3;9(6):e98753. doi: 10.1371/journal.pone.0098753 (PMC4043794; doi:10.1371/journal.pone.0098753)
Supplement: Table S1 — Secondary structure composition of GdnHCl/urea formed fibrils as determined from deconvolution and curve fitting of the FTIR amide I band. (PDF) [file pone.0098753.s003.pdf]

Table S1. Secondary structure composition of GdnHCl/urea formed fibrils as determined from deconvolution and curve fitting of the FTIR amide I band.

**Assignment**	**Wavenumber (cm^{-1})**	**Secondary structure (%)**
Intermolecular β-sheet	1616	16
Intermolecular β-sheet	1627	22
β-pleated sheets	1637	16
α-helix	1650	19
Turn	1662	19
Turn/loops	1672	15
Turn	1683	9.6
